# Ex Vivo Preconditioning as a Useful Tool for Modification of the Extracellular Matrix of Multipotent Mesenchymal Stromal Cells

**DOI:** 10.3390/ijms26136301

**Published:** 2025-06-30

**Authors:** Elena Andreeva, Olga Zhidkova, Diana Matveeva, Aleksandra Gornostaeva, Margarita Lobanova, Ludmila Buravkova

**Affiliations:** Institute of Biomedical Problems, Russian Academy of Sciences, 123007 Moscow, Russia; matveeva.dajana@yandex.ru (D.M.); hindiii@yandex.ru (A.G.); pogodina_m@mail.ru (M.L.); buravkova@imbp.ru (L.B.)

**Keywords:** multipotent mesenchymal stem/stromal cells (MSCs), in vitro preconditioning, hypoxia, inflammatory mediators, 3D culture, scaffolds, pharmacological agents, extracellular matrix, regenerative medicine and tissue engineering

## Abstract

Cell technologies have provided promising tools for modulating the properties of multipotent mesenchymal stem/stromal cells (MSCs) to meet the needs of cell therapy as well as tissue engineering and regenerative medicine (TERM). Ex vivo preconditioning is directed at enhancing the engraftment of MSCs and activating their secretory activity, primarily the production of soluble mediators. The present review aims to highlight the underestimated effect of the most accepted preconditioning approaches on the modification of the important set of insoluble molecules secreted by MSCs into extracellular space—the extracellular matrix (ECM). A thorough review of the published literature was performed, with particular emphasis on ECM-related data. The analysis of data on ECM changes showed that most of the applied preconditioning methods—hypoxia, inflammatory priming, pharmacological agents, 3D culture, and scaffolds—generally stimulate ECM production, increase the deposition of growth factors, promote alignment, and increase ECM stiffness. There are already preliminary results demonstrating the successful application of preconditioned ECM for promoting angiogenesis, targeted stromal lineage differentiation, and other therapeutic goals. The prospects for further research in this area are discussed.

## 1. Introduction

Multipotent mesenchymal stem/stromal cells (MSCs) are a highly sought-after product for regenerative medicine [1,2,3]. These cells can be readily isolated from diverse tissues and expanded in vitro, both to amplify and to modulate their activity in a desired direction [4,5,6,7]. Targeted modulation of MSC function is referred to as preconditioning, licensing, or priming and has been widely used in protocols of ex vivo MSC preparation for clinical needs, as comprehensively reviewed in several recent papers [7,8,9,10,11,12].

Preconditioning has been demonstrated to effectively modulate the inherent properties of MSCs and their derivatives, namely soluble mediators and exosomes [11,13]. The application of cell-free products is regarded as a superior alternative to using cells due to its capacity to mitigate the risk of an immune response from the recipient. As a result, the ability to regulate the secretory activity of MSCs is in high demand [14,15,16,17]. A range of preconditioning strategies to modulate the profile of secreted mediators from MSCs has been proposed, contributing to enhanced anti-apoptotic, regenerative, anti-scarring, immunomodulatory, and angiogenic effects.

**Figure 1:**
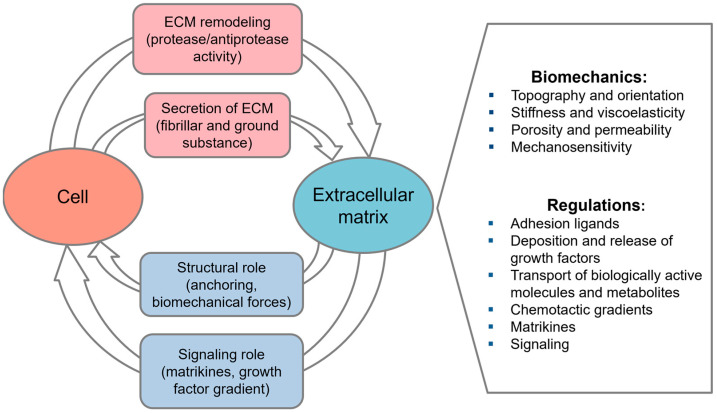
Bidirectional cell–matrix interaction in local milieu.

The important set of insoluble molecules secreted by MSCs into extracellular space—the extracellular matrix (ECM)—has also garnered increasing interest. The ECM is comprised of insoluble fibrillar proteins, including collagens, elastin, and laminins, along with soluble glycoproteins and proteoglycans that collectively form hydrogels, occupying the space between the fibers. The heterogeneity of matrix molecules and their synergistic interplay are pivotal in determining the biomechanical and regulatory functions of the ECM within diverse tissue environments [18,19,20,21,22,23,24,25,26] (Figure 1).

Multipotent mesenchymal stem/stromal cells (MSCs) and their stromal lineage progeny are considered the primary producers of extracellular matrix (ECM) in the body. A number of studies have described the effects of decellularized ECM (cell-derived ECM (cd-ECM)) generated by MSCs. After recellularization of these matrices with MSCs, the newly applied cells demonstrated improved adhesion [27,28], enhanced proliferation, and migration [27,29,30,31,32]. In the presence of specific stimuli, the tree-lineage differentiations in osteo- [27,30,31], adipo- [27,32], and chondro- [33] directions were increased. The MSC cd-ECM as a substrate has been used for the maintenance of the naive phenotype of progenitor cells: hematopoietic stem and progenitor cells [34], embryonic stem cells [35], neuronal progenitors [36,37], and MSCs, including the reduction in spontaneous osteo-commitment [29,31,38,39].

It is reasonable to hypothesize that the implementation of preconditioning may result in alterations in the characteristics of both the MSCs and the ECM produced by them. The objective of this review was to systematize the available data on ECM modulation under well-established MSC preconditioning protocols with the aim of identifying the potential for applications of such matrices in the field of tissue engineering and regenerative medicine (TERM).

## 2. Cell-Derived Extracellular Matrix

ECM from decellularized tissues has emerged as a prominent biomaterial in clinical applications [40]. Nevertheless, there are a number of challenges associated with the use of such matrices. The limited availability of human tissues and possible ethical issues can only be partially compensated for by animal sources due to the possibility of xenogeneic incompatibility. Furthermore, the inherent composition of tissue matrices precludes modulation for specific applications. Additionally, incomplete decellularization could result in unwanted immune responses [41].

In light of the aforementioned points, decellularized matrices produced by cells in vitro (cd-ECM) have garnered significant attention. These constructs can be derived from suitable cell types, exhibiting a composition that closely resembles that of their “parental” tissues [42]. Through prolonged cultivation, it is feasible to obtain a substantial quantity of cd-ECM, which exhibits characteristics analogous to those of the ECM in native tissue. Very promising results have already been demonstrated in the application of scaffolds made of cd-ECM in the field of tissue engineering. Ex vivo, a key benefit is the ability to standardize cells before their application and to assess their safety. Cell manipulation enables precise control over cd-ECM properties, directing tissue engineering outcomes [43]. Controlled decellularization preserves the biological activity of the cd-ECM and reduces immunogenicity. This approach enables the production of ECM with specific properties, making cd-ECM a versatile universal material for physiological studies and TERM applications [44].

At the same time, it is important to emphasize the challenges related to the manufacturing and characterization of cd-ECM. The key factors affecting the clinical translation of cd-ECM can be outlined as follows.

The cultivation protocol aiming to obtain ECM needs to be adopted for certain cell types, including the growth media recipes, culture duration, and decellularization methods in order to maximize the structure and integrity of the cd-ECM in 2D and 3D settings. Traditional decellularization approaches include chemical and physical methods, while emerging techniques, such as the induction of cellular apoptosis, are gaining attention [45,46]. In addition, it is necessary to develop a set of parameters characterizing the composition and physical properties based on various microscopic and biochemical approaches, including proteomic approaches [21,45]. It is also necessary to optimize evaluation of the efficacy of decellularization. Quantification of DNA in the samples and microscopy-based studies (for example, fluorescence, atomic force, and scanning electron microscopy) could be used. For further clinical use, it is necessary to select xeno-free and chemically defined culture media and systems for cd-ECM isolation in sterile conditions [21,47].

Special attention needs to be given to residual immunogenicity of cd-ECM samples. After decellularization, certain antigens—including major histocompatibility complex (MHC) molecules and minor histocompatibility antigens—may remain. Fragmented ECM components like heparan sulfate, low-molecular-weight hyaluronic acid, elastokines, biglycan, decorin, and fibronectin can act as damage-associated molecular patterns (DAMPs) [48,49,50]. Certain structural proteins, like vimentin, are naturally antigenic and can therefore contribute to the immunogenicity of decellularized tissues [51]. DAMPs are identified by immune cell receptors such as TLR, PR, RAGE, P2X7, CD44, and NKG2D, acting as adjuvants for both antigen-nonspecific and antigen-specific immune responses. This leads to the activation of immune cells, the release of inflammatory cytokines, and the formation of inflammasomes [48]. Elevation of inflammatory cytokines also causes a shift in the polarization of macrophages as a critical factor determining the long-term prognosis of transplants towards the M1 phenotype [52,53]. Overall, excessive decellularization is undesirable because it removes essential structural proteins, depleting the matrix and compromising its functional properties. For instance, the loss of collagen IV, fibronectin, or laminin can impair angiogenesis [54]. Therefore, decellularization protocols must prioritize the reduction in immunogenicity. Notably, a method that combines mechanical disruption with alpha-galactosidase treatment has demonstrated high effectiveness, successfully removing cells and xenoantigens while maintaining essential biochemical components [55]. Intensive crosslinking, particularly using chemical agents, reduces the biodegradability of ECM and results in a higher M1/M2 macrophage polarization ratio, leading to milder inflammation and foreign body response compared to non-crosslinked counterparts after implantation [56,57,58]. A novel antigen removal method employing human recombinant alpha-galactosidase is being developed to reduce the immunogenicity of xenogenic ECM [59].

Finally, the critical issue is standardization of CD-ECM for the TERM application. Tissue-derived ECM products have already been approved for clinical applications [47]. Cd-ECM samples have not been studied sufficiently for clinical use. Certain aspects require standardization, including decellularization efficiency metrics, sterility testing protocols, and specifications for biomedical properties. Meanwhile, the positive therapeutic outcomes of the cd-ECM application have been demonstrated in vitro and in animal models [60].

### 2.1. Cell-Derived ECM as a Physiological Microenvironment

Stromal lineage cells, including MSCs and fibroblasts, are most commonly used to obtain cd-ECMs under standard 2D or 3D culture conditions. These ECM products can be applied in vitro for reseeding with different cell types or in various TE constructs.

At present, cd-ECMs are used in a variety of applications. Coatings for cell culture plasticware are the most commonly used, as the matrix structures create a microenvironment that is more physiologically appropriate than conventional culture plasticware or single ECM proteins. This enables the preservation of cell phenotype and activity, which is particularly important for stem and progenitor cells, known to lose their self-renewal capacity and to undergo senescence in vitro [29,61,62]. It has been shown that cd-ECM from MSCs can reproduce the stem cell niche sufficiently to protect reseeded MSCs from oxidative stress, promote their proliferation, and maintain their self-renewal capacity [29,38,63,64]. Such MSC-derived cd-ECM can effectively maintain the native phenotype of neuronal progenitor cells [36], embryonic stem cells [35], periodontal ligament stem cells [39], and hematopoietic stem cells [34]. In addition, cd-ECM generated from young MSCs has been found to rejuvenate already aged MSCs [27,65,66]. These effects are closely related to the biological profile of the extracellular matrix [45].

### 2.2. Tissue-Specific Memory: Instructive Role of Cell-Derived ECM

ECM produced by MSCs has been shown to exhibit distinct patterns in its content and structure, contingent on the tissue source of the MSCs [60]. This has prompted research interest in the preservation of such ECM tissue-specific memory following decellularization and subsequent recellularization. The phenomenon of tissue-specific differentiation of reseeded MSCs has been well-documented in the literature. In particular, cd-ECMs derived from cartilage, tendon, and bone marrow MSCs (BM-MSCs) promoted the differentiation of reseeded MSCs into cartilage, tendon, and bone lineages, respectively [67,68,69]. Furthermore, a study by Rao et al. (2014) demonstrated the induction of a myofibroblast phenotype in MSCs seeded on cd-ECM derived from smooth muscle cells [70]. In addition to their parental tissue-specific protein profiles, differently sourced cd-ECMs exhibit differences in stiffness (as determined by atomic force microscopy). For instance, cd-ECM derived from BM-MSCs has been shown to exhibit a stiffness index 3-fold higher than those derived from adipose-tissue-sourced MSCs (AT-MSCs) [30]. Prewitz et al. (2013) conducted an analysis to compare the stiffness indices of cd-ECM from BM-MSCs, embryonic and neonatal skin fibroblasts, and umbilical vein endothelial cells [34]. Their findings indicated that cd-ECM from BM-MSCs exhibited the most robust embodiment of elasticity and stiffness parameters, exhibiting similarity to native bone marrow.

These data are consistent with the hypothesis that, in vivo, MSCs are localized in areas exhibiting distinct biophysical properties such as matrix stiffness, topography, and adhesion ligand density on ECM structures [71]. The topology of the matrix is perceived by a variety of cellular receptors. These receptors transmit the extracellular signals from the ECM to the cytoskeleton and, further, to the nucleo-skeleton and chromosomes. This results in changes in gene expression and cell functions depending on the properties of the substrate [72]. This conversion from mechanical signals to biochemical signals underlining the cellular response is known as mechanotransduction [73]. As demonstrated in previous studies, the stiffness of the ECM can modulate the differentiation of MSCs. For instance, adipogenic and neural differentiation have been found to be stimulated on soft substrates, while myo-, chondro-, and osteogenic differentiation are induced on rigid coatings [74,75]. Hoshiba and Lu (2011) have observed that chondrocyte adhesion is more effective on chondroblast-derived cd-ECM than on cd-ECM from dermal fibroblasts or MSCs [76]. Concurrently, a number of studies have demonstrated that less-related cd-ECM generated from synovial fluid, umbilical cord, and BM-derived MSCs or from fibroblasts can have positive effects on the proliferation of chondroblasts [32,77,78]. The observed stimulation of proliferation may be attributed to the elevated fibronectin content in the MSC and fibroblast ECM, as compared to that in chondrocyte-derived ECM [76].

These data could be of significance in the development of tissue-engineered constructs for cartilage repair. In vitro, chondrocytes are known to undergo rapid dedifferentiation; therefore, reproducing native conditions with chondroblast-derived cd-ECM can effectively support chondrocyte commitment [77]. Consequently, cd-ECM can be utilized to establish tissue-specific niches to support or enhance cellular functions and to investigate cell–niche interactions in depth. Beyond this, there is also considerable demand for cd-ECM and its derivatives in a variety of clinical applications.

### 2.3. Cell-Derived ECM in Tissue Engineering and Regenerative Medicine

The high biological activity of cd-ECM, as evidenced by in vitro experiments, has served as a background for the development of protocols for the use of such cd-ECM in clinical settings. The application of cd-ECM in TERM is gaining prominence, both as a standalone modality and for providing the components of biomaterials. Three-dimensional scaffolds composed entirely of cd-ECM have been obtained by decellularizing multilayer cell sheets [79] and spheroids [80] or by depositing ECM onto supporting materials such as hollow tubes/fibers [81] and porous scaffolds [82].

Currently, the use of cd-ECM-based biomaterials is predominantly focused on the fields of skeletal and cardiovascular repair. However, a range of additional applications is being contemplated, including potential uses in skin regeneration [83] and peripheral nerve repair [84].

For applications requiring biomaterials with specific mechanical properties, cd-ECM can be integrated with synthetic materials to create hybrid scaffolds [85,86]. These hybrid materials have been shown to meet the necessary mechanical requirements and to provide adequate biochemical stimulation, thereby facilitating implant integration and functionality [87]. Typically, cd-ECMs are used to generate coatings by simple removal of the cells from the biomaterial surface [88,89] or by applying previously dissolved cd-ECM [90]. As an alternative, cd-ECM components can be incorporated directly into biomaterials during synthesis, for instance, by electro-spinning [85,86].

### 2.4. Targeted Modification of Cell-Derived ECM

As discussed earlier, a key advantage of cd-ECM over tissue-derived ECM is its adjustability, which can be realized through various approaches.

It is possible to achieve biochemical re-engineering of the ECM through direct effects on the matrix-producing cells. Such approaches may include genetic modifications as reported by Higuchi et al. (2010) [91], altered culture conditions and medium compositions: reduction of O_2_—hypoxia, serum deprivation, ascorbate supplementation, and other exogenous factors [45,92,93]. It is also possible to modify already synthesized ECM structures during post-translational modification. This can be achieved by introducing certain functional groups into the ECM molecules, leading to changes in the matrix properties. As an illustration, azide-modified monosaccharides added to the culture medium subsequently became incorporated into the ECM [94]. In this way, the cd-ECM can be covalently linked to the surfaces of biomaterials [95]. Furthermore, ECM stiffness can be modulated through post-processing techniques, such as cross-linking [96].

Mechano-physical reorganization is an additional instrument for ECM modification. The structural and compositional characteristics of cd-ECM are profoundly influenced by the substrates on which the producer cells are cultured. These substrates can include micro- and nano-structured surfaces [97,98,99], hydrogels [100], and synthetic carriers (micro-molds) [101]. The alterations in such biochemical and mechano-physical properties of the cd-ECM are able to turn on feedback loops, resulting in changes in gene expression and the behavior of reseeded cells [97,102].

Thus, modern methods of cell biology offer a wide range of approaches to target the stromal lineage progenitors, primarily MSCs, to stimulate the production, and to modify the functional activity of the ECM they produce. Below, we highlight how the most common MSC preconditioning protocols for the needs of cell therapy and TERM affect the properties of MSC-derived ECM.

## 3. MSC Preconditioning for Improvement of Cell Therapy and TERM-Demanded Properties: What About the Extracellular Matrix?

In the meantime, significant progress has been made in ex vivo strategies to enhance the potential of MSCs for clinical applications. These approaches are referred to as MSC preconditioning, also known as priming or licensing.

**Figure 2:**
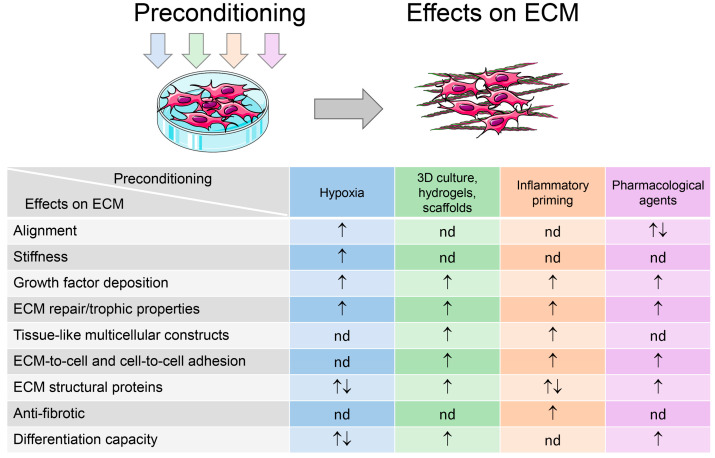
Preconditioning tools for improvement of multipotent mesenchymal stem/stromal cells’ (MSC) properties for cell therapy and tissue engineering and regemerative medicine (TERM) applications and extracellular matrix (ECM)-related outcomes. ↑—indicates the stimulation of certain function; ↓—indicates the attenuation of certain function; nd—not determined. The artwork used in this figure was adapted from Servier Medical Art (https://smart.servier.com/ (accessed on 16 June 2025)). Servier Medical Art by Servier is licensed under a Creative Commons Attribution 3.0 Unported License.

Preconditioning affects both the MSCs themselves and their derivatives. Preconditioning is aimed at ensuring the homing of MSCs to target tissues by increasing their resistance to harmful factors at the site of damage, for example, through reducing sensitivity to oxidative stress and, therefore, apoptosis, as well as by enhancing migration. The second task is associated with boosting production of bioactive mediators, primarily anti-inflammatory and angiogenic mediators [11,13] (Figure 2).

The diverse beneficial effects of preconditioning MSCs that allow their successful application in cell therapy and TERM are discussed in detail in a number of modern reviews [7,8,9,10,11,12,103,104,105,106]. Bibliometric analysis of publications on MSC priming and preclinical (clinical) outcomes [9] has suggested a list of the most common, successful preconditioning protocols. These are hypoxia, bioactive mediators (e.g., inflammatory cytokines, growth factors, and hormones), and pharmacological agents, together with control of topographical and other culture conditions (2D vs. 3D). We shall next consider the effects of these approaches in the context of the nature of the ECMs that can be produced by MSCs.

### 3.1. Hypoxia

Presently, hypoxic preconditioning constitutes one of the most common approaches for in vitro modification of MSCs for cell therapy and for TERM. The underlying reason for this is that, during cell therapy, upon introduction into sites of injury, MSCs are faced with a hostile microenvironment, including hypoxia [107]. In addition, tissues that have been damaged are poorly vascularized and thus are restricted in their ability to support the metabolic processes of the implanted MSCs. This, in turn, can lead to their rapid apoptosis after transplantation. It is hypothesized that MSCs that have typically been expanded in standard CO_2_ incubators under ambient air conditions may lack the capacity to effectively adapt to the harsh milieu. On the other hand, hypoxic preconditioning has been shown to enhance the stability and therapeutic potential of these cells [7,8,9,10,11,12]. In order to create a hypoxic environment in vitro, multigas incubators or sealed chambers with pre-established oxygen levels are utilized [108].

A number of studies have demonstrated that hypoxic preconditioning stimulates an increase in proliferative activity while attenuating the response to differentiation stimuli. Furthermore, hypoxic preconditioning also stimulates an increase in the production of soluble mediators, primarily angiogenic ones [109,110,111]. These changes appear to be related to the increased contribution of glycolysis to ATP production [112].

It is reasonable to suggest that hypoxic priming may have an effect on the structural, biochemical, biophysical, and, most significantly, instructional properties of the ECM.

Cell adaptation to O_2_ deprivation occurs through the hypoxia-sensitive signaling pathway by activation of hypoxia-inducible transcription factors (HIFs) [113]. Within cells, HIF-dependent regulation of numerous genes is observed, including those involved in glycolysis, angiogenesis, the cell cycle, apoptosis, development, and differentiation [114]. Gornostaeva et al. (2024) have recently reviewed in detail the changes in the structural and regulatory molecules within the ECM under hypoxia, including the mechanisms of downstream target gene regulation by transcription factors HIF-1, -2, and -3 [115].

In the present review, we have focused on describing the modulation of the extracellular matrix during hypoxic conditioning of MSCs, performed to enhance their therapeutic efficacy. Here, we consider both short hypoxic exposures, which induce a rapid response of the MSCs at the level of gene and protein expression, and longer exposures to highlight the resulting structural changes to the ECM (Table 1).

Proteomic analysis of both BM-MSCs and their extracellular vesicles after 48 hours’ exposure at 1% O_2_ revealed decreased quantities of collagens and regulatory glycoproteins. Enrichment analysis of the reactome pathway for proteins altered in hypoxic BM-MSCs and extracellular vesicles has identified pathways related to glycosaminoglycan metabolism in extracellular vesicles, ECM organization, including elastin fibers, and the degradation of chondroitin sulfate/dermatan sulfates [118]. Mass spectrometry revealed an enrichment of AT-MSC lysates and conditioned medium (CM) after exposure to 1% O_2_ with lysyl oxidase, prolyl hydroxylase, and prolyl oxidase. These enzymes are involved in the resistance to tension, protein folding, and mechanical stability of collagen fibrils [119,120]. After 10 days of exposure to hypoxia, MSCs from joints show a significant increase in metalloproteinases and their inhibitors, which could indicate activation of ECM remodeling [121]. However, long-term expansion of AT-MSCs under hypoxia, such as at 5% O_2_, does not lead to excessive ECM accumulation [124,125].

Thus, the ECM of hypoxia-primed MSCs can undergo significant alterations. Next, we shall consider the potential therapeutic applications of hypoxia-modulated ECM.

As mentioned above, hypoxic MSCs exhibit increased angiogenic activity [128,129,130,131,132]. Changes in the ECM after the hypoxic preconditioning of stromal cells may be interesting from the perspective of therapeutic angiogenesis. Both structural and regulatory proteins of the matrisome are known to participate in angiogenesis [133,134,135,136,137]. The interaction of endothelial cells with the ECM, mediated by integrins, triggers complex signaling cascades that regulate their survival, proliferation and migration, and, as a result, blood vessel stabilization. Furthermore, it has been demonstrated that ECM-deposited growth factors and matrikines—fragments of ECM structural proteins—possess both pro- and antiangiogenic activity [138]. Subcutaneous implantation of a nanoporous collagen scaffold coated with ECM from fibroblasts exposed to hypoxia for 10 days induced a directed angiogenic response within one week [139]. It was showed that, after hypoxic preconditioning, the proteomic profile of exosomes from dental-pulp-derived MSCs was enriched with proangiogenic proteins. The majority of these proteins included thrombospondin-1, perlecan, and fibulin-1, as well as the regulatory enzymes LOXL-2 and MMP-2 [12].

It is well established, that exosomes can act as mediators of the ECM either through direct EV–ECM interactions or by influencing cell–ECM interactions. In turn, ECM regulates the secretion and uptake of exosomes. Thus, altered exosome cargo could modulate significantly the ECM properties [140,141].

In the review by Sylakowski et al. (2020), it was suggested that the use of hypoxic MSCs reseeded onto cd-ECM or multicellular sheets of hypoxic MSCs on their own ECM could be effective in ischemic conditions in vivo, particularly in chronic wound therapy protocols [107]. Such constructs are expected to increase MSC survival rate as well as to enhance their proangiogenic potential. It is further hypothesized that the use of cd-ECM from hypoxia-preconditioned MSCs may be more efficacious due to the combination of structure-forming proteins and deposited growth factors. For instance, an enhancement in wound healing was observed in a skin defect model in mice using cd-ECM from MSCs conditioned by CuCl_2_, a hypoxia mimetic. Furthermore, the enrichment of ECM with types I and III collagens, as well as the growth factors TGF-β1, VEGF, and FGF-2, was detected in these cd-ECMs, promoting enhanced granulation tissue formation, angiogenesis, and rapid re-epithelialization [122]. Hypoxic preconditioning has been demonstrated to upregulate VEGF and to stimulate its encoded protein, which is one of the master growth factors in angiogenesis [142]. Buczek-Thomas demonstrated that short-term hypoxia (1% O_2_ for 48 h) resulted in an increased VEGF level in the ECM of retinal pigment epithelium [135]. This effect was associated with alterations in the structure of heparan sulfate proteoglycans [135]. Furthermore, glycosaminoglycans and proteoglycans may influence the deposition of other proangiogenic molecules, particularly FGF-2 [49,143]. Kim and Ma have demonstrated that MSC-derived cd-ECM at 20% and 5% O_2_ exhibited diverse capabilities to bind FGF-2, a positive regulator of angiogenesis. Furthermore, the cd-ECM from MSCs under hypoxia exhibited a 2-fold elevation in bound FGF-2 as well as a superior capacity to absorb exogenous FGF-2 [127]. This finding indicates the potential efficacy of ECMs derived from hypoxic MSCs during the early phases of wound healing, when active vascular growth is required.

Another potential application of ECM from hypoxic MSCs is its incorporation into tissue-engineered constructs such as those used for cartilage replacement [144,145,146]. It is well established that cartilage development is associated with low oxygen levels [147,148]. In vitro studies have shown that physiological hypoxia can promote the production of cartilage-specific matrix components in MSCs, including unsulfated glycosaminoglycans (GAGs), chondroitin-4-sulfate, aggrecan, and collagen types II, IX, and XI [149,150,151]. Our own preliminary data have demonstrated that MSC expansion under physiological hypoxia results in spontaneous enrichment of their matrisome with cartilage-specific collagens (types XII and XIV) and proteoglycans (*BGN*, *FMOD*, *HAPLN1*, and *CILP*), which participate in GAGs metabolism, contribute to the elastic properties of the ECM, and participate in the organization of collagen fibers. It can be hypothesized that the hypoxia-dependent enrichment of the ECM with GAGs may provide a biochemical advantage for the use of hypoxic ECM in tissue-engineered constructs.

Long-term cultivation of chondrocytes at 4% O_2_ results in a significant HIF-1α-dependent upregulation of *LOX*, accompanied by an increase in the cross-links between collagen fibrils and an increase in ECM stiffness [126]. After 11 days at 2% O_2_, it has been observed that BM-MSCs exhibit an upregulation of HIF-2α, along with increased alignment of fibronectin fibrils [123]. A similar alignment has been demonstrated in ECM produced by MSCs under physiological hypoxia (5% O_2_) [28]. The increased alignment of fibrils can serve as an indirect indicator of enhanced ECM stiffness, a factor that has been demonstrated to promote the differentiation toward the chondrogenic lineage and the maintenance of chondroblast expansion on cd-ECM from hypoxia-primed MSCs.

Several studies have demonstrated that permanent low O_2_ levels in vitro more closely mimic physiological tissue conditions. An O_2_ of 1–5% is not hypoxic for many cell types but rather represents in situ normoxia, often referred to as physiological hypoxia [152]. As we have described earlier, continuous cultivation under physiological hypoxia ensured significant modification of MSC functions manifested in increased proliferative potential, attenuated response to differentiation stimuli, and increased angiogenic activity [109,111]. These changes appeared to be related to the increased contribution of glycolysis to ATP production [112]. Based on these observations, we supposed that ECM turnover and structure may be affected as well. A comprehensive comparative analysis of cd-ECM derived from MSCs cultured at either 20% or 5% O_2_ has been conducted in our laboratory [28]. The hypothesis of this study was that, under physiological hypoxia, the ECM derived from MSCs would retain a “hypoxic memory”, which would manifest as the induction of a hypoxic phenotype in reseeded MSCs further expanded under ambient O_2_. The study has successfully demonstrated the phenomenon of the MSCs featuring an O_2_-level-dependent “ECM-educated” phenotype. This was reflected in both a change in paracrine profile and in their attenuated osteo-commitment potential. The ability of the ECM to preserve the microenvironmental competence of the MSCs and to “educate” the surrounding cells makes it a potentially effective tool for the fields of cell therapy and tissue engineering.

It can thus be concluded that the topology and protein composition of ECMs induced by physiological hypoxia are promising with regard to the use of cd-ECM as biomimetics of the tissue microenvironment in various TERM applications.

### 3.2. Tissue Engineering Approaches (3D Culture, Hydrogels, and Scaffolds)

Three-dimensional (3D) cultivation and the use of different types of biomaterials for cultivation have been demonstrated to provide viable methods of MSC priming. The field of 3D approaches can be roughly divided into two categories: scaffold-free and scaffold-based methods. MSCs in either three-dimensional scaffolds or aggregates are known effectively to possess enhanced transcription and paracrine secretion of a number of proangiogenic, anti-apoptotic, anti-inflammatory, and immunomodulatory factors. Furthermore, such 3D cultured cells have also been shown to demonstrate enhanced therapeutic effects, a phenomenon that could partly be attributed to altered ECM protein production [153,154,155,156,157,158].

The effect of 3D conditions on the ECM properties has received comparatively less attention than have other influences on MSC activities. Consequently, it would be a valid approach to pose an inquiry into how the composition and the deposition of ECM proteins are modified by 3D conditions. The available data are summarized in Table 2.

In the absence of carriers or scaffolds, MSCs can be cultured as 3D spheroids, clumps, or cell sheets [172]. The most common carriers used include natural compounds such as alginates, collagen, chitosan, gelatin, and hyaluronic acid, as well as synthetic biodegradable meshwork based on polymers (polylactic acid (PLA), polyglycolic acid (PGA), polyethylene, and polypropylene) [172,173].

In multicellular constructs, integrins and intercellular adhesion molecules are characterized by their elevated expression, thereby enhancing cell–cell and cell–matrix interactions. In contrast to 2D culture, where cells spread out and become clearly oriented, 3D culture enables cells to receive additional mechanical signals from the local environment. Mechanosensitive pathways transmit signals to the nucleus, thereby affecting the transcription profile of each cell [174,175,176].

Under 3D conditions, the altered transcription of many matrix-associated genes has been demonstrated in stromal lineage cells (MSCs and fibroblasts) [159,161,162]. In comparison with traditional 2D approaches, 3D culture induces upregulation of genes encoding collagens of different types, fibronectin, laminin, etc. (Table 2). It is of note that this effect has been described for stromal cells and fibroblasts from different tissues [159,161,162]. Meanwhile, the expression levels of other matrix-associated genes in these cells differed, probably depending on variations in the culture conditions and tissue sources. Indeed, as previously shown, MSCs from different sources retain their ability to produce tissue-specific ECM molecules in vitro [170,177,178].

In comparison with the 2D setting, 3D cultivation enhances not only transcription but also the release of ECM proteins into extracellular space [169]. As Clément et al. demonstrated through proteomic analysis, 3D expansion can be accompanied by increases in the total amount of secreted proteins as well as of ECM-associated molecules within 12 [161].

Multicellular aggregates in vitro have generated a fine peculiar 3D network of ECM proteins. These proteins have been detected immunocytochemically as early as day 3, with subsequent, continued accumulation monitored for a longer period of 11–28 days of cultivation [168]. MSCs within such aggregates have been observed to produce higher quantities of collagens of types I, II, III, IV, V, and VI, as well as significant quantities of fibronectin and laminin, in comparison to two-dimensional cultures [154,159,160,161,164,165,169,171,179] (Table 2). In terms of therapeutic applications, the multicellular aggregates have the potential to demonstrate superiority over suspended MSCs [154,171]. This is likely attributable to the preservation of natural cell–ECM interactions across different types of multicellular constructs. For example, such proliferation is known to be regulated by a number of ECM molecules such as collagen and fibronectin [180]. These molecules have been shown to enhance cell adhesion and viability, crucial considerations when MSCs are introduced into recipient tissues [181]. In addition, the ECM scaffold appears to protect the multicellular construct once it has been introduced into the recipient’s tissue [154].

Upon lineage-restricted induction, the upregulation of corresponding key transcriptional factors, such as Runx2 and Sox9, and the accumulation of lineage-specific ECM proteins have been detected in MSC aggregates [182,183]. Furthermore, the accumulation of collagen V, laminin, perlecan, and osteopontin, which it has been claimed are required for chondrogenesis, neuronal growth, and osteogenesis, respectively, has been demonstrated in the ECM of 3D MSC constructs [184,185,186]. Elevated expression of adipose-tissue-specific collagen IV has also been detected upon adipogenesis in MSC aggregates [164,187].

The stiffness and topographical features of ECMs have been demonstrated to regulate the differentiation potential of MSCs via mechanotransduction [74,188,189,190]. Specifically, in softer alginate gels, there was an upregulation of genes encoding cartilage proteins, including *COL1A1*, *COL2A1*, *COLX*, and *ACAN*, along with increased production of collagen II [191] and GAGs [165,166,167,192]. The addition of GAGs to the alginate gel has been shown to enhance this effect [165]. By contrast, the cultivation of MSCs on stiffer porous carriers has been shown to activate the expression of genes encoding bone-specific ECM proteins [193]. Additionally, the modification of substrate topography, for instance, through the formation of pores or protrusions, promotes the secretion of ECM proteins by MCSs [191,193,194].

There are remaining several unresolved issues that slow down the clinical application of 3D-cultured cd-ECM constructs. Three-dimensional cultivation encompasses many different approaches, which complicates the standardization of the resulting products [195,196]. Depending on the cultivation method, cellular aggregates can vary greatly in size and structure. Smaller aggregates tend to exhibit lower biological activity. Moreover, O_2_ and nutrient diffusion is uneven in large multicellular aggregates [197], which can affect the composition of the resulting 3D cd-ECM.

To mitigate nutrient deficiency in the deeper layers of multicellular aggregates, it is essential to optimize cell plating density and employ cultivation methods that minimize size variability [197,198]. For clinical applications, scaffolds with well-defined stiffness, porosity, and biochemical composition are preferred. Therefore, polymeric materials used in tissue engineering must be fully characterized [173].

Standard analytical methods developed for 2D surfaces may not be suitable for 3D structures. Atomic force microscopy, magnetic microrheometry, and volume electron microscopy are promising tools for assessing the physical characteristics of 3D matrices, including stiffness, viscosity, and surface nanotopography [199,200,201,202].

Insufficient decellularization of cd-ECM increases the risk of immunological reactions in the recipient [48,49]. Additionally, the nanotopographical architecture of polymeric scaffold surfaces can hinder cd-ECM coverage and reduce the efficiency of scaffold decellularization [203]. Therefore, decellularization protocols should be specifically optimized for 3D cd-ECM constructs. Despite the limited clinical application, 3D models are of interest for studying the pharmacological effects of various substances, for analyzing the biomechanical properties of polymer materials, as well as for studying the mechanisms of cell migration and communication.

The combined application of 3D settings and culture medium supplements, such as differentiating stimuli or others, is of particular interest for TERM protocols. Thus, MMC, achieved by supplementing synthetic or naturally derived molecules that induce excluded volume effects, has been shown to effectively mimic the physiological cellular environment during matrix secretion. It has been demonstrated that MMC with ficoll, carrageenan, or dextran has the capacity to enhance the expression of ECM molecules, including laminin, fibronectin, collagen I, and GAGs. The MMC approach was also provided decellularized ECM, in which laminin was organized as fibrils [160].

The positive outcomes of 3D culture conditions are likely due to their provision of a more authentic cell culture setting compared to 2D protocols. The 3D conditions generate a more realistic physiological microenvironment, thus inducing cell responses that are more reminiscent of in vivo conditions [204,205,206].

The field of combining biomaterials with 3D cultures represents a particularly valuable resource for tissue engineering. The preconditioning of cells that occurs when using 3D cultivation on various substrates has emerged as a useful approach for the generation of decellularized ECM possessing advanced characteristics. The different resulting matrices can then be used for the repair of specific tissues rich in ECM, such as skin, muscles, and bones.

### 3.3. Inflammatory Microenvironments

Inflammatory priming is a commonly used approach for MSC preconditioning, aiming to improve the immunosuppressive and anti-inflammatory properties of the MSCs. For this purpose, factors secreted by activated immune cells, such as TNF-α, IFN-γ, and IL-1β, or recombinant analogs are used [207,208]. The secretion of IDO, PGE2, and other mediators has been shown to be stimulated in MSCs in response to such priming. Depending on the priming cytokine used, the composition of the immunomodulatory cocktails generated by the MSCs can be controlled. For instance, IFN-γ has been observed to stimulate the secretion of IDO, while TNF-α induces PGE2 [163,208,209].

Further experiments have demonstrated that, in addition to the stimulation of immunomodulation, inflammatory cytokines also affect other functional aspects involving MSCs, including the metabolism of the ECM. For instance, upregulation of the genes encoding PEDF, VEGF, CSF2, and others has been demonstrated in response to such preconditioning [208]. Furthermore, enhanced secretion of various growth factors by MSCs has been observed [115,210,211]. The growth factors and other mediators secreted by MSCs have been shown to bind with structural components of the ECM, including fibronectin, decorin, and tenascin-C [211]. This binding results in the deposition of these factors into the ECM [212,213,214]. This may consequently affect the properties of the ECM.

In addition, it has been demonstrated that inflammatory preconditioning exerts a direct influence on ECM structural components as well as on ECM regulatory molecules (see Table 3).

IFN-γ priming of MSCs has been observed to result in the upregulation of genes for the expression of adhesion molecules, as well as the upregulation of genes for the alpha and gamma chains of fibrinogen [163]. TGF-β1 has been observed to induce upregulation of genes for the matrix structural components, integrins and MMP-2, while MMP-1 expression was downregulated [220]. TNF-α has been shown to upregulate *BMP2* and to induce encoding BMP-2 production. In addition, TNF-α induced osteogenic differentiation of MSCs via the Erk1/2 MAPK signaling pathway [215]. Furthermore, IL-1β priming led to upregulation of genes encoding MMPs, adhesion molecules, and integrins. It is thought that a significant contribution to this effect is derived from NF-κB signaling through IL-1β, which is known to promote phosphorylation of NF-κB [210,216]. GO analysis of down- or upregulated MSC genes after IL-1β priming has enabled the identification of specific pathways involved in the modulation of inflammation and ECM remodeling [217]. The treatment of MSCs with a combination of IFN-γ, TNF-α, and IL-1β has been shown to result in an upregulation of the genes encoding MMPs [208]. The conditioned medium (CM) of MSCs primed with a combination of IFN-γ and TNF-α acquires an antifibrotic pattern, with elevated levels of DKK1, follistatin, cathepsin S, and the ECM-remodeling MMP-1 and -3. DKK1, an antagonist of the Wnt/β-catenin signaling cascade, binds to LRP6 co-receptors and promotes the hyperphosphorylation of β-catenin, inducing its degradation. Follistatin is known to suppress the TGF-β canonical pathway and collagen I expression. Cathepsin S is involved in TGF-β activation and, through the endothelial protein C receptor, inhibits endothelial-to-mesenchymal transition [221].

As is the case with immunomodulation, various cytokines exert disparate effects on ECM production and turnover. For instance, IFN-γ priming has been shown to upregulate *ICAM1*, while *COL10A1* and *COL3A1* were downregulated. In contrast, IL-17 stimulation has been observed to trigger upregulation of MMPs, accompanied by downregulation of *ITGA6* [219]. Furthermore, after preconditioning with TNF-α, several MMP genes in MSCs were found to be upregulated, whereas only *MMP1* was found to be upregulated after IFN-γ stimulation [209].

The concentrations of such priming cytokines are also important factors to consider. Li et al. have demonstrated that priming MSCs with low levels of TGF-β1 (0.1 ng/mL) significantly upregulated genes encoding major ECM proteins but downregulated *MMP1*, while TGF-β1 at 1 ng/mL upregulated only *COL1A1*. Conversely, higher concentrations of TGF-β1 (up to 10 ng/mL) lead to a substantial inhibition of the transcription of genes for laminin and integrin beta 5 and to the upregulation of MMPs [220].

The MSC-CM derived from IFN-γ and TNF-α primed MSCs has been shown to have antifibrotic properties in lung explant cultures, resulting in remodeling of the ECM. Here, the levels of collagen I and fibronectin decrease, indicating a reduction in fibrotic activity [221]. It is important to note that the properties of the CM may vary depending on the composition of the priming cocktail. In a study by Jammes et al., the effect of CM from primed BM-MSCs on articular chondrocytes in 3D culture was examined. Their findings indicated that MSC-CM, after IL-1β and TNF-α priming, has the capacity to stimulate the production of cartilage-specific collagen types I and IIB in chondrocytes. The effect was more pronounced with the IL-1β priming. Conversely, following IFN-γ stimulation, MSC-CM provoked a suppression of collagen production. Furthermore, the study revealed that the transcription of protease-encoding genes was upregulated in CM from IL-1β-primed MSCs, while these genes were downregulated in CM from IFN-γ-primed MSCs [222]. The therapeutic efficacy of CM from IL-1β-primed MSCs has also been demonstrated in an ex vivo model of bovine intervertebral discs (IVDs), suggesting a potential clinical application. The genes encoding pro-inflammatory cytokines were found to be downregulated, while *MMP3* and *TIMP2* were found to be upregulated [218].

In general, priming of MSCs with inflammatory mediators may promote antifibrotic changes in the ECM secreted by these MSCs and enhance their repair/trophic properties due to the deposited cytokines. However, it should be kept in mind that the effects depend on the type of cytokine and its concentration as well as the tissue source of the MSCs. In addition, there are certain challenges that would be faced if the inflammatory-induced ECM were to be used in TERM. The immunogenicity of such ECMs is one of the major limitations. The deposition of inflammatory factors within the ECM upon MSC priming is another disadvantage also because of potential inflammatory reactions [48]. In addition, MSCs themselves can release inflammatory mediators in response to priming. Notably, it has been shown that IL-1β preconditioning of MSCs increases the secretion of the inflammatory agents IL-6, IL-8, MCP-1, etc. [218,222]. When MSCs were pre-conditioned with TNF-α, the levels of IL-6, CXCL8, and CXCL10 were also shown to increase [222].

Thus, the successful application of inflammatory priming for ECM modulation requires the development of preconditioning protocols and the selection of cocktails with particular compositions and concentrations of soluble factors to obtain ECMs with the desired properties.

### 3.4. Pharmacological Agents and Growth Medium Composition

Currently, the attention of researchers is also attracted to MSC preconditioning by various pharmacological agents that are being actively used to improve the regenerative potential of MSCs, including their angiogenic, immunosuppressive, and other trophic activities [7]. It can be assumed that these agents will influence the quality and quantity of the ECM and affiliated molecules produced by the MSCs (Table 4).

The effects of some pharmacological agents on MSCs lead to alterations in their metabolic pathways that may then influence the synthesis and regulation of the ECM. For instance, agents such as the hypoxia mimetics 2,4-dinitrophenol, isoflurane (ISO), dimethyloxalylglycine (DMOG), and cobalt chloride (CoCl_2_) have been shown to increase the expression of HIF-1 in MSCs both at the gene and protein levels [233,234,235]. As discussed above, HIF-1 transcription factor governs the organization and remodeling of the ECM.

Macromolecular crowding, as noted previously, is a process entailing the addition of large inert molecules (e.g., fucoidan, carrageenan, and dextran sulphate) to the cultivation medium of MSCs, with the objective of augmenting ECM production. The consequence of this process is an acceleration of ECM synthesis, in addition to an increase in fibril alignment (of type I collagen, fibronectin, and laminin) [229,230].

In MSCs, the stimulatory effect of heparin on the transcription of osteogenic genes, as well as the enhancement of osteogenic differentiation after lipopolysaccharide treatment has been demonstrated [237,238].

Ascorbic acid and its derivatives are the most widely used inducers of ECM production. The increase in collagen and elastic fibers in the ECM of human dental-pulp-derived MSCs has been demonstrated [224]. After recellularization of such ECM with fresh MSCs, the enhanced adhesion, proliferation, and both osteogenic and chondrogenic differentiation has been described [226,227]. Yi et al. noted that preconditioning of mouse BM-derived MSCs with ascorbic acid 2-glucoside promotes upregulation of *HIF1A* and *VEGF* as well as enhancing collagen production [225].

In MSC differentiation media, not only does the formation of ECM components specific for certain mesenchymal lineages occur but the ECM structure itself also undergoes changes. For instance, S. Pérez-Castrillo et al. compared the structure of ECM obtained in the presence of ascorbate and in chondrogenic medium. The latter case exhibited a denser matrix, with a more orderly distribution of aligned fibrils, as well as the presence of cartilage-specific aggrecan and hyaluronates. Irrespective of the tissue source (adipose or bone marrow), the ECMs from MSCs following chondro-induction provide enhanced support for the viability and proliferation of recellularized MSCs [223].

As with cytokine priming, in order to modify the properties of the ECM, it is important to consider the concentrations of the pharmacological agents employed. To illustrate this point, retinoic acid has been shown to increase both the formation of mineralized matrix and total collagen synthesis in MSCs, but only at low concentrations [228].

Therefore, modifying the composition of the culture media, including by the addition of pharmacological agents, represents a promising approach that allows modulation of the microenvironment of the MSCs, alteration of their physiological activity, and the accumulation of ECM in predictable ways necessary for use in TERM. It is logical to assume that such modification should result in changes of ECM function. However, the details of this have not been fully demonstrated. Further studies are needed to clarify the nature of these changes.

### 3.5. Applications of ECM Derived from Preconditioned MSCs and Their Progeny

Various applications have been investigated for cd-ECM, including fundamental research, pathophysiological studies, and TERM [60]. The findings related to the use of cd-ECM derived from preconditioned MSCs and their more differentiated progeny are limited, mainly presented by in vitro experiments and preclinical studies (Table 5).

In vitro studies have demonstrated that cd-ECM derived from adipo-, chondro-, or osteo-induced MSCs promoted similar differentiation pathways in newly seeded MSCs [224,226,227,239,240]. After ectopic implantation in mice, cd-ECM from osteo-differentiated MSCs were shown to support bone formation more effectively, than cd-ECM from undifferentiated MSCs [241]. Maintaining hypoxic conditions during expansion significantly enhanced regenerative capacity of cd-ECM from MSCs and dermal fibroblasts, with studies demonstrating improved therapeutic efficacy in tissue regeneration models [242,243].

Most available data have been obtained in experiments in which cells were pre-cultured on various coatings: polycaprolactone (PCL) scaffolds and fibers [86,89,244,245], chitosan–silk fibroin scaffolds [84,246], PCL/silk fibroin scaffold [247], biocompatible polyvinyl alcohol (PVA) hydrogels [83], Poly(Lactic-co-Glycolic Acid) (PLGA) nanofibers [248], and Poly(L-lactide-co-glycolide)/poly(L-lactide) scaffolds [102]. The positive effects of cd-ECM mobilization have been demonstrated in various preclinical models, such as wound healing [83,248], cardiac remodeling after myocardial infarction (MI) [249], bone formation [250], nerve defect regeneration [84,246,251], and hindlimb ischemia [252].

A review of the existing preclinical data clearly indicates that preliminary modification of ECM through preconditioning of parent cells is likely to be in great demand, broadening the range of novel materials available for TERM.

**Table 5:** Application of modified cd-ECM in in vitro and in vivo studies.

Cell Type	Preconditioning/ Modification	In Vivo/In Vitro Outcomes	Reference
**Pharmacological agents and culture medium formulations**			
huBM-MSCs	Osteogenic medium	cd-ECM from osteo-differentiated MSCs promoted bone formation more effectively after ectopic implantation in mice, than cd-ECM from undifferentiated MSCs	[241]
huAT-MSCs	Adipogenic medium	cd-ECM from adipo-differentiated MSCs induced adipogenic differentiation of reseeded MSCs in vitro	[239]
rat BM-MSCs	Biphasic calcium phosphate scaffolds	Increased osteoblastic differentiation of reseeded cd-ECM coated BCP scaffolds in vitro	[240]
**Hypoxia**			
rbBM-MSCs	Chemical hypoxia, CoCl_2_	Hypoxic cd-ECM accelerated wound repair in a mouse model of full-thickness skin defect (enhanced reepithelization and granulation tissue formation, augmented angiogenesis)	[242]
huDF	2% O_2_, in combination with polycaprolactone (PCL) scaffold and mechanical stimulation	Hypoxic PCL-cd-ECM patches improved endothelization and smooth muscle regeneration after grafting in rat abdominal aorta	[243]
**Scaffolds and coatings**			
murine osteoblast/osteocyte-like cells	Porous PLC scaffolds	Cd-ECM-coated scaffolds induced cell proliferation, osteogenic activity in vitro, and potentiated angiogenesis in chorioallantoic membrane assay in ovo	[244]
huBM-MSCs	Porous PCL scaffolds	Cd-ECM-coated scaffolds enhanced attachment, proliferation, and osteogenic differentiation of reseeded MSCs	[245]
rat fibroblasts and endothelial cells (ECs)	PCL microfibers	Cd-ECM-coated microfibers stimulated tube formation by ECs, osteoblast proliferation, and differentiation	[253]
huBM-MSCs, HUVEC	PCL microfibers	Enhancement of osteogenic differentiation of reseeded MSCs on Cd-ECM-coated microfibers	[89]
huAT-MSCs	Poly(Lactic-*co*-Glycolic Acid) (PLGA) nanofibers	Cd-ECM-coated nanofibrous mesh improved the wound healing in a mouse skin wound model	[248]
human lung fibroblast (hLF)	Polyvinyl alcohol (PVA) hydrogel	Cd-ECM incorporated in PVA hydrogel provided advanced skin regeneration in infected wound mice model	[86]
huDF	PVA hydrogel	Cardiac remodeling was improved in the infarcted area of the rat MI model with a cardiac patch that included MSCs seeded on cd-ECM incorporated in poly(vinyl alcohol) (PVA) hydrogel	[249]
hLF	PLGA/PLA-based scaffolds	Cd-ECM coated scaffolds stimulated reseeded MSC osteo-differentiation. Significant increase in new bone formation in a mouse ectopic and rat calvarial bone defect models.	[250]
rat BM-MSCs	Chitosan-silk fibroin scaffolds	Enhancement of nerve regeneration in rat model of peripheral nerve injury	[84]
huBM-MSCs	Chitosan/silk fibroin scaffolds	Cd-ECM-coated grafts significantly improved nerve repair in dog sciatic nerve gap model	[246]
huBM-MSCs	Chitin/chitosan fibers	Cd-ECM-coated fibers induced the repair of sciatic nerve defects in rats similar to autografts	[251]
hLF	Collagen hydrogel	Microspheres containing HUVECs, MSCs and cd-ECM incorporated in collagen hydrogel significantly improved blood reperfusion in a mouse hindlimb ischemic model	[252]

## 4. Conclusions

Decellularized matrices derived from cultured MSCs are attracting increasing interest from both academic researchers and clinicians. This is due to a number of properties of such cd-ECM that are in demand for the recreation of tissue niches and the elucidation of fundamental mechanisms of bidirectional cell-matrix regulation, as well as for the increasing demand for use in TERM. In this regard, the focus on the effects of MSC preconditioning on their ECM properties seems to be very timely.

Meanwhile, in the available sources focused on preconditioning protocols for MSCs, matrix modification is garnering practically no attention as a certain outcome of priming. At the same time, analysis of the existing literature data has shown that, in different preconditioning settings, the properties of the ECMs are noticeably changed, and the resulting ECMs can significantly differ in their biological activity from the ECMs of native MSCs (Figure 2).

As discussed above, hypoxic preconditioning has been observed to activate enzymes that crosslink the ECM components. This activation has been shown to result in alterations to the fibril packing characteristics of the produced ECM, accompanied by fiber alignment and an increase in their stiffness. Furthermore, ECM becomes enriched with viscoelastic glycosaminoglycans and deposited proangiogenic growth factors. These changes are regarded as highly promising for their potential applications in the engineering of tissue chondro-explants, as well as in the context of therapeutic angiogenesis.

The 3D multicellular constructs (spheroids, cell sheets, and clumps) have been demonstrated to significantly enhance the expression of matrix-associated genes and proteins in MSCs. The structural distribution of ECM molecules during 3D culture tends to assemble into supramolecular aggregates, as occurs in vivo. Furthermore, 3D cultivation facilitates the generation of tissue-like constructs that closely resemble the composition of bone or cartilage tissues, which is in demand for replacement therapy of the disorders.

Preconditioning of MSCs by pharmacological agents has been shown to exert a number of effects. These include an increased production of the ECM, alterations in its composition and structure, and an enhancement of the viability, adhesion, proliferation, and differentiation potential of MSCs in osteo- and chondrogenic directions following recellularization.

Despite the diverse molecular mechanisms involved in achieving preconditioning effects, they often result in similar manifestations. Figure 2 highlights several key preconditioning outcomes reported in the available literature. In all cases except hypoxia, the increase in ECM production and improvement of cell-to-ECM and cell-to-cell adhesion is described. For inflammatory conditioning only, there are no data on the impact on differentiation. In other cases, both inducing (3D culture and pharmaceutical agents) and bidirectional (hypoxia) effects are demonstrated. Exposure to hypoxia and some pharmacological agents leads to the alignment of ECM structures, which significantly affects its mechanical properties. Importantly, all preconditioning techniques increase the deposition of growth factors, modulate ECM structural proteins, and enhance its regenerative potential.

The possibility of producing matrices with new functional activity as a result of preconditioning opens up wide prospects for further research in the field of RM. It is possible to delineate several principal domains in which “preconditioned” ECM may be in demand. Preconditioned cd-ECM can be utilized as a substrate not exclusive to MSCs but can be extended to various cell types such as to endothelial cells, augmenting their angiogenic potential, and to fibroblasts, stimulating their regenerative capacity. An important characteristic of cd-ECMs is their ability to retain a memory of the properties of those cells that produced them, as well as to transfer this memory to newly applied cells [246]. Solubilized cd-ECMs can be used as a bioactive mediator and bioactive supplements to culture media or in clinical protocols [247]. The current focus of interest is the employment of MSCs within diverse cellular constructs incorporating ECM. These types of constructs include multilayered cell sheets, spheroids, and scaffolds, among others. Preconditioning enables precise, targeted modulation of both the characteristics of the MSCs and their ECM, thereby enhancing the effectiveness of MSC-based TERM protocols [83].

The introduction of preconditioning has the potential to significantly enrich the range of cd-ECM biomechanical engineering methods. This enrichment would result in obtaining biomaterials with defined and improved properties. Additionally, the development of interdisciplinary strategies for engineering cd-ECMs is crucial, encompassing not just biological manipulation but also integration with other disciplines such as materials science. To achieve this objective, it is essential to develop improved methodologies and innovative interdisciplinary approaches to create a new generation of cd-ECMs for basic research and to develop protocols for their clinical applications in TERM.

## Figures and Tables

**Table 1:** The effects of hypoxic preconditioning on extracellular matrix and its regulatory molecules.

MSC Type	Experimental Condition	Effect	Reference
huBM-MSCs	1% O_2_, 24 h	↑ *P4HA1*, *MMP9*, *TIMP3*, *VEGFA*, *PGF*	[116]
huBM-MSCs	1.5% O_2_, 48 h	↑ *COLXV*	[117]
rat BM-MSCs	1% O_2_, 48 h	↓ collagens (type I, III, XIV), fibrillin-2, fibulin-1, laminin-5, osteonectin, ECM1, TIMP2 Enrichment analysis of reactome pathway identified pathways related to glycosaminoglycan metabolism, extracellular matrix organization, including elastin fibers, and degradation of chondroitin sulfate/dermatan sulfates	[118]
huAT-MSCs	1% O_2_, 24 h	↑ collagens I and III types and cross-linking enzyme *LOX*, *LOXL2*, *P4HA1*, *P4HA2*, *PLOD2*, *PLOD1*	[119]
huAT-MSCs	1% O_2_, 48 h	↑ *LOXL1*, *LOXL2*, *LOXL3*, *PLOD1*, *PLOD2*, *TIMP1*	[120]
huDP-MSCs	2% O_2_, 48 h	↑ trombospondin-1, perlecan, fibullin-1, LOXL-2, MMP-2 GO enrichment analysis identified specific pathways involved in regulation angiogenesis	[12]
huBM-MSCs	5% O_2_, 3 days	= membrane-bound or secreted MMP activity in MSC-CM	[121]
rbBM-MSCs	CuCl_2_–hypoxia mimetic, 7 days	↑ collagen of type I and III, TGF-β 1, VEGF-α, FGF-2 deposition in ECM	[122]
huBM-MSCs	2% O_2_, 7 days	↑ collagen I type Hypoxia-related increase in the alignment of fibronectin fibrils	[123]
huBM-MSCs	5% O_2_, 10 day	↑ *MMP9*, *MMP10*, *MMP11*, *MMP12*, *TIMP1*, *TIMP3*	[121]
huAT-MSCs	5% O_2,_ 14 days	↑ Alignment of fibrillar structures and stiffness. cd-ECM from physiological hypoxia is able to ensure the maintenance of the low-commitment state of MSCs	[28]
huAT-MSCs	5% O_2_, 14 days	= deposition of collagen and non-collagen proteins under the MSC monolayer	[124]
huBM-MSCs	2% O_2_, 7 days or 14 days	= collagen I and III types, fibronectin, laminin, MMP activity, expression of *COL1A1* and *P4HA1*	[125]
AC calves	4% O_2_, 3 weeks	↑ *LOX*. ↑ number of cross-links between ECM fibrils and stiffness	[126]
huMSCs	2% O_2_, 15 days	↑ deposition of collagen IV type and laminin = deposition fibronectin and vitronectin in cd-ECM ↑ deposition of FGF-2	[127]

↑—increase, ↓—decrease, =—no changes.

**Table 2:** Tissue engineering approaches as a tool for modification of ECM compartments in cultured MSCs.

MSC Type	Experimental Condition	Effect	Reference
huAT-MSCs	MSCs were cultured as 3D multicellular aggregates using the hanging droplet method, 7 days	↑ *SDC1*, *SDC2*, *BGN*, *COL8A2*, *COL14A1*, *COL15A 1*, *COL18A1*, *COL6A3*, *FNDC3A*, *LAMA1*, *LAMB1*, *TIMP1*, *TIMP3*, *MMP1*, *MMP2*, *MMP8*, *MMP9*, *TNC*. ↑ MMP-2, MMP14, TIMP-1, tenascin C, collagen VI α3, fibronectin 1.	[159]
huUCB-MSCs	3D spheroid culture, macromolecular crowding, 48 h	MSCs deposited ECM proteins, including collagen type I, fibronectin, and laminin. Macromolecular crowding application to MSC spheroid cultures facilitate ECM assembly in a 3D configuration.	[160]
huDF	Cells were cultured in form of fibroblast sheet, 28 days, 50 μg/mL AA	↑ *FNDC1*, *CILP*, *CLIP2*, *IBSP*, *THBS4*, *COL4A3*, *COL14A1*, *COL24A1*, *COL6A5*, *COL10A1*, *SPON1*, *SERPINA5*, *GPM6B*, *LAMC3*, *GPC3*. ↓ *MATN2*, *SPP1*. Quantitative proteomic profile: ↑ 74 matrisome-related proteins and ↓ 35 matrisome-related proteins in exosomes from 3D cultures. ↑ MMP-1, MMP-2, MMP-3, MMP-8, MMP-9, MMP-13, TIMP-1, TIMP-4 in 3D-exosomes.	[161]
huBM-MSCs	Dynamic 3D culture as cellular spheroids, 7 days	↑ *PRG4 SPP1 SPON1 COL24A1*. ↓ *ACAN*, *COL11A1*, *CSPG4*.	[162]
huMSCs from nasal turbinate tissue	3D spheroid culture, 3 days	↑ fibronectin and laminin.	[163]
huAT-MSCs	3D spheroid culture, 9 days	↑ fibronectin, collagen type V, VI.	[164]
huBM-MSCs	3D culture in porous alginate foams supplemented with chondroitin sulfate, 14 days	MSC produced an ECM containing sGAGs and types II and I collagen	[165]
huUC-MSCs	3D culture in alginate hydrogel with hyaluronic acid, 28 days	↑ *ACAN*, *COL2A1*, ↑ collagen II	[166]
huBM-MSCs	3D culture in alginate hydrogel with HA, 28 days	↑ *COLX*, *COMP*, ↑ collagen X	[166]
huAF-MSCs	3D culture in phenolated alginate-collagen hydrogel, 21 days	↑ *COL2A1*	[167]
huUC-MSCs	3D cultures as cellular spheroids, 4–11 days	Cells produced ECM composed of mainly collagen I, fibronectin, laminin, and collagen IV	[168]
rb synovial MSCs	3D spheroid culture, hanging drop culture, 14 days	↑ *SPP1*, *TFPI2*	[169]
huUC-MSCs, huAT-MSCs, huDP-MSCs	3D spheroid culture, 21 days	↑ *ACAN*, *COL2AI*	[170]
rat BM-MSCs	MSC bodies in methylcellulose hydrogel, 24 h	MSC produced collagen type I, type III, fibronectin, and laminin in cell bodies	[154]
huAT-MSCs	3D spheroid culture, hanging drop culture, 3 days	↑ collagen I, fibronectin, and laminin	[171]

↑—increase, ↓—decrease.

**Table 3:** ECM-related effects of inflammatory priming of MSCs.

MSC Type	Experimental Condition	Effect	Reference
huBM-MSCs	TNF-α, 25 ng/mL, 48 h	↑ *MMP1*, *MMP3*, *MMP10*, *MMP12*	[209]
huBM-MSCs	IFN-γ, 25 ng/mL, 48 h	↑ *MMP1*	[209]
huAT-MSCs	TNF-α, 1 ng/mL, 3 days	↑ BMP-2	[215]
hu-gingival MSCs	IL-1β, 1 ng/mL, 24 h	↑ MMP-1, MMP-9	[216]
huAT-MSCs	IL-1β, 1 ng/mL, 48 h	GO enrichment analysis of up- or downregulated genes identifying specific pathways involved in the modulation of inflammation and extracellular matrix remodeling	[217]
huBM-MSCs	Cocktail IFN-γ, TNF-α, and IL-1β, 20 ng/mL, 10 ng/mL, and 20 ng/mL, 24 h	↑ *IL6*, *IL8*, *CXCL10*, *CCL2*, *IDO1*, *COX2*, *VEGFA*, *FGF2,* and *MMP2*	[208]
huBM-MSCs	IL-1β, 10 ng/mL, 48 h	Primed MSC-CM: ↓ *IL6*, *IL8* ↑ *MMP1*, *MMP3,* and *TIMP2* ↑ aggrecan deposition in degenerative IVD	[218]
huBM-MSCs	IFN-γ, 30 ng/mL, 20 h	↑ ICAM-1 and VCAM-1 ↑ *FGA*, *FGG*	[163]
huBM-MSCs	IL-1β, 25 ng/mL, 24 h	↑ collagen, fibronectin, laminin ↑ *MMP1*, *MMP3*, *ICAM1,* and *ICAM4*	[210]
huBM-MSCs	IFN-γ, 500 U/mL, 5 days	↓ *ICAM1*, *COL10A1*, *COL3A1*, *COL1A1*, *ADAM12*	[219]
huBM-MSCs	IL-17A, 50 ng/mL, 5 days	↑ *MMP13*, *MMP1* ↓ *ITGA6*	[219]
huUC-MSCs	TGF-β1, 0.1 ng/mL, 24 h	↑ *COL1A1*, *COL4A4*, *FN1*, *ITGB5*, *TNC*; ↓ *MMP1* ↑ collagen I, collagen IV, fibronectin, integrin beta 5, and tenascin-C, MMP-2; ↓ MMP-1	[220]
huUC-MSCs	TGF-β1, 1 ng/mL, 24 h	↑ *COL1A1*, *MMP1*, *MMP2*, *MMP9*; ↓ *LAMA1*, *ITGB5* ↑ collagen I; ↓ laminin and integrin beta 5	[220]
huUC-MSCs	TGF-β1, 10 ng/mL, 24 h	↑ *COL1A1*, *MMP1*, *MMP2*, *MMP9*; ↓ *LAMA1*, *ITGB5* ↑ MMP-1; ↓ laminin and integrin beta 5	[220]
huAT-MSC-CM	IFN-γ, 10 ng/mL + TNF-α, 15 ng/mL, 72 h	Primed MSC-CM: ↓ fibrogenic myofibroblasts ↑ ECM remodeling ↓ collagen I and fibronectin ↓ fibrotic load in TGF-β treated lung explant cultures ↑ antifibrotic proteins DKK1, MMP-1, MMP-3, follistatin, cathepsin S	[221]
eqBM-MSC-CM	IL-1β, 20 ng/mL, 24 h	↑ *MMP1*, *MMP13*, *HTRA1*, ↑ collagen types IIB and I in eAC 3D cultures	[222]
eqBM-MSC-CM	TNF-α, 10 ng/mL, 24 h	↑ collagen (types IIB and I) accumulation in eAC 3D cultures	[222]
eqBM-MSC-CM	IFN-γ, 100 ng/mL, 24 h	↓ collagen (types IIB and I), ↓ *HTRA1* and *MMP13*	[222]

↑—increase, ↓—decrease.

**Table 4:** The influence of pharmacological agents and culture medium formulations on ECM and ECM-related molecules in MSCs.

MSC Type	Experimental Condition	Effect	Reference
huAT-MSCs	Chondrogenic medium, 14 days	↑ ECM density ↓ Ordered ECM structure + aggrecan and hyaluronates ↑ Viability and proliferation of MSCs after recellularization	[223]
huAT-MSCs	AA, 50 µM, 15 days	↓ ECM density Uneven fibers, randomly distributed	[223]
huBM-MSCs	Chondrogenic medium, 14 days	↑ ECM density ↑ Ordered structure + aggrecan and hyaluronates ↑ Viability and proliferation of MSCs after recellularization	[223]
huBM-MSCs	AA, 50 µM, 15 days	↓ ECM density ↑ Homogeneous ECM structure and porosity	[223]
huDP-MSCs	AA, 50 µg/mL, 14 days	↑ *COL6A1*, *COL6A2*, *COL6A3*, *FBN1*, *FBLN2*, *FN1*, *HSPG2* In fibroblasts during osteogenic induction after recellularization: ↑ *ALP*, *RUNX2*, *OCN* ↓ *COL1A1*	[224]
huDP-MSCs	Osteogenic medium, 21 days	↑ *ANXA1*, *ANXA4*, *ANXA5*, *ANXA6*, *ANXA7*, *ANXA11* In fibroblasts during osteogenic induction after recellularization: ↑ Accumulation of calcium and phosphate, ALP ↑ *ALP*, *RUNX2* ↓ *COL1A1*	[224]
huBM-MSCs	AA- 2-glucoside 0.74 mM, 3 days	↑ *HIF1*, *VEGF*	[225]
huAT-MCSs	AA, 5 µM, 50 µM, 250 µM, 500 µM, 10 days	↑ Matrix stiffness and chondrogenic potential	[226]
huDP-MSCs and periodontal ligament	AA, 250 µM, 7 days	↑ Adhesion, proliferation, and osteogenic differentiation of MSCs from dental pulp after recellularization	[227]
huDP-MSCs	RA, 0.1 µM, 1 µM, 10 µM, 1, 3, 7, 14 days	↑ Mineralized matrix formation and collagen synthesis at concentration 0.1 µM	[228]
huDP-MSCs	AA, 3 µM, 30 µM, 300 µM, 1, 3, 7, 14 days	↑ Mineralized matrix formation and collagen synthesis at concentrations 30 and 300 µM.	[228]
MSCs	Fucoidan, 100 µg/mL, 7 days	↑ ECM synthesis and osteogenic differentiation of MSCs ↑ Alignment of ECM fibers	[229]
huUC-MSCs	Carrageenan, λ medium viscosity, 10–50 µg/mL, 4 days	↑ Deposition of collagen types I, III, and IV, fibronectin and laminin	[230]
huBM-MSCs	Icariin, 1 × 10^−6^ M, 14 days	↑ ECM synthesis Encoding genes: ↑ *COL2A1*, *ACAN*, *SOX9* Protein levels: ↑ *COL2A1*, *ACAN*, *SOX9*	[231]
huDP-MSCs	CCPA, 15–60 nM, 8 days	↑ *RUNX2* and *ALP* on the 3rd and 7th day of exposure, ALP activity on the 7th day and ECM mineralization after 21 days	[232]
huBM-MSCs	2,4-dinitrophenol, 0.25 µM, 20 min	↑ *VEGF*, *HIF*, *KIND3*, *CD29*, *CD44*	[233]
huBM-MSCs	DMOG, 1 µM, 24 h	↑ HIF-1α, VEGF, Glut-1	[234]
huBM-MSCs	ISO 1%, 2%, 2, 4 h	↑ *CXCR4*, *HIF1A* ↑ CXCR4, HIF-1α	[235]
huBM-MSCs	Diazoxide, 200 μM/L, 30 min	↑ *FGF*, *HGF*	[236]
huAT-MSCs	LPS, 0.1 µg/mL, 14 days	↑ Proliferation and osteogenic differentiation of MSCs	[237]
huAT-MSCs	Heparin 1.3 IU/mL, 13 IU/mL, 14 days	↑ *BMP2*, *BMP6*, *ALPL*, *RUNX2*, *BGLAP*, *SMURF1*	[238]

↑—increase, ↓—decrease.

## Data Availability

Not applicable.

## Abbreviations

The following abbreviations are used in this manuscript:

MSCs	multipotent mesenchymal stem/stromal cells
TERM	tissue engineering and regenerative medicine
ECM	extracellular matrix
cd-ECM	cell-derived ECM
TE	tissue engineering
RM	regenerative medicine
BM	bone marrow
AT	adipose tissue
DP	dental pulp
UCB	umbilical cord blood
UC	umbilical cord
AF	amniotic fluid
DF	dermal fibroblasts
DP	dermal papilla
CM	conditioned medium
GAGs	glycosaminoglycans
sGAGs	sulfated glycosaminoglycans
PGA	polyglycolic acid
MMC	macromolecular crowding
PLA	polylactic acid
HIF	hypoxia-inducible transcription factor
ISO	isoflurane
DMOG	dimethyloxalylglycine
CCPA	2-chloro-N6-cyclopentyl-adenosine
AA	ascorbic acid
RA	retinoic acid
HA	hyaluronic acid
Hu	human
Rb	rabbit
Eq	equine
eAC	equine articular chondrocytes
IVD	bovine intervertebral disc
